# Intelligence Beyond Crime: AI, EI, and the Quest for Work-Life Balance in Policing

**DOI:** 10.12688/f1000research.170760.1

**Published:** 2026-04-30

**Authors:** Dr. Neha Dubey, Meenu Shant Priya, Ashutosh Pandey, Mohit Yadav, Mohd Asif Shah

**Affiliations:** 1 Management, Galgotias University School of Business, Greater Noida, Uttar Pradesh, 203201, India; 2 FORE, School of Management, New Delhi, India; 3 JGBS, O.P. Jindal Global University, Sonipath, India; 4 Visiting Scholar, International School, Vietnam National University, Hanoi, Vietnam; 5 Management, Kardan University, Kabul, Kabul, 1007, Afghanistan; 6 Division of Research and Development, Lovely Professional University, Phagwara, Punjab, 144001, India; 7 University Centre of Research & Development, Chandigarh University, Gharuan, Mohali, Punjab, 140413, India

**Keywords:** Police; Artificial Intelligence; Emotional Intelligence; Work-Life Balance; Organizational Commitment; Bibliometric Review; Systematic Literature Review

## Abstract

In recent years, the ideas of emotional intelligence and artificial intelligence have gained a lot of traction in the relevant literature. This study investigates the impact of artificial intelligence (AI) and emotional intelligence (EI) on the work-life balance (WLB) of police personnel through a comprehensive secondary data analysis. Utilizing bibliometric analysis, the research systematically and critically examines existing literature on intelligence and WLB. Based on 91 research papers sourced from the Scopus database, the study analyzes publication trends, theoretical foundations, highly referenced publications, and journals, frequently applied keywords, and key topics of inquiry within prominent clusters. Additionally, a thematic overview of the Intelligence and the Work-life Balance body of work developed through bibliographic coupling is provided. Content analysis of recent publications highlights potential research gaps and emerging trends. The findings reveal a blend of established and evolving research themes, broadening knowledge of the useful effects of AI and EI on work-life balance in law enforcement. This article stands out as the first to employ diverse bibliographic mapping techniques, offering a broad perspective on the Intelligence and WLB corpus while suggesting Possible recommendations for further investigation.

## Introduction

Work-life balance is a critical issue that impacts employees’ productivity and well-being in a wide range of enterprises. Work-life balance is the capacity to strike a balance between the demands of one’s profession and personal life, including family. Work-life balance is the capacity of an individual to equitably allocate their time and energy between their personal and professional lives. Some sources explain the balance between work and personal life in a number of ways. This balance is defined as the capacity to effectively integrate work, family, and personal responsibilities, as per Greenhaus and Beutell ( Minarika et al., 2020). Maintaining equilibrium between one’s work and many aspects of life has become a major public concern in recent years as more workers realize the need for what is now referred to as “work-life balance” ( Kumari and Kataria, 2022).

Goleman (1998) popularized the idea of emotional intelligence (ELB) in the workplace using this phrase, WLB. Emotional intelligence (EI) includes the ability to monitor one’s own and others’ emotions, distinguish between various emotions and assign them the proper labels, and use emotional information to inform decisions and actions. Employees and organizations in communication roles benefit most from EI ( Yao et al., 2019).

Emotional intelligence and organizational capabilities have been widely examined across management and behavioral research. Prior studies have linked emotional intelligence to individual and organizational outcomes ( Mayer and Salovey, 1993; Barsade and Gibson, 2007a, b; Pradhan et al., 2016; Kataria, 2021), while strategic and dynamic capability perspectives highlight the role of managerial and organizational competencies in performance and decision-making ( Amit and Schoemaker, 1993; Sirmon et al., 2007; Mikalef et al., 2020a, b; Dwivedi et al., 2021). Recent studies further emphasize the growing role of digital transformation and artificial intelligence in shaping organizational processes and work outcomes ( Montoya and Rivas, 2019; Nuryanto, 2021; Deloitte, 2023; Fauzi et al., 2023; Ahmad et al., 2023; Ezzeddine et al., 2023; Dubey et al., 2025).

According to Russell and Norvig (2016), machine intelligence is another name for artificial intelligence (AI). After the 1950s, it became a popular topic in scholarly literature. The fields of communication, information technology, health, agriculture, logistics, education, and aviation all make use of AI. In the banking, human resources, health, tourist, and hotel industries, in particular, it generates revenue. Today, many experts believe that artificial intelligence, and machine learning in particular, can help people, cities, and even countries in numerous ways. It has been suggested, for instance, that so-called machine learning may soon relieve workers of some of their responsibilities, which might enhance workflows or assist in identifying the most efficient methods of completing jobs ( Pawlicka A, P. M., 2020).

The Nature of contemporary labor has been drastically altered by the introduction of intelligence (EI & AI) into the workplace, which presents previously unheard-of chances for productivity, creativity, and expansion.

The literature on integrating emotional intelligence (EI) with artificial intelligence (AI) is summarized in this review, along with its implications for organizational efficiency. WLB is especially important in high-stress occupations like law enforcement, where balancing work and family obligations can be difficult. According to recent studies, police personnel can benefit from the integration of artificial intelligence (AI) and emotional intelligence (EI) in managing the demands of their jobs ( Viegas & Henriques, 2023). These observations highlight how crucial it is to strike a long-term balance between work and life, especially in the complicated and demanding workplaces of today. Moreover, there is still a dearth of comprehensive literature outlining how to address the influence of AI and EI on work-life balance. A bibliometric examination of the literature on intelligence (EI, AI) and work-life balance resulted from the paucity of research on which practices and procedures, particularly quantitative approaches, may be used to promote the formation of EI and EI ( Robertson et al., 2012; Simeone et al., 2020).

Therefore, This introductory section establishes the fundamental principles for a comprehensive examination of Intelligence (EI & AI) and Work-life balance, including its theoretical underpinnings, real-world uses, and the continuous changes in its execution. The extensive body of research on cognitive ability and work-life balance has already been thoroughly researched, covering a wide range of classifications, fundamental notions, parameters, and variables. However because there is a lack of an institutional structure, the available literature in this field appears dispersed, necessitating integration to improve the synthesis of current research and spur fresh discoveries. This study attempts to condense the body of existing research on work-life balance along with intelligence (EI & AI) and provide a detailed view of the same through a systematic literature review (SLR) and various bibliographic mapping approaches. Existing reviews in Intelligence (EI & AI) & Work-life balance did not consider bibliometric analysis to provide a comprehensive overview of the research in the area. This study is unique in the sense that it aims to map the evolution of the existing research patterns, identify the current research dynamics, and assess the emerging research paradigms in the field. Providing an update on the state of research on intelligence and work-life balance is the primary goal of our review paper. The following questions outline the study’s scope:

RQ1:
Which articles about intelligence (EI & AI) and work-life balance are the most recent each year?

RQ2:
How often are articles about intelligence and work-life balance (EI & AI) published per Subject Area?

RQ3:
What are the most popular subjects for intelligence (EI & AI) and work-life balance in the future?

For the purpose of helping academic researchers better understand the current state of research in the fields of intelligence (EI & AI) and work-life balance, an analysis of publication patterns organized by number of years, affiliated countries, journals, authors, and economy type has been accomplished.

In the following respects, this study advances the topic of work-life interface:

(1) Earlier evaluations of work-life balance and intelligence (EI & AI) did not take bibliometric analysis into account to provide a thorough overview of the field.
(2) This study also aims to provide future research directions by analyzing the content of articles that have been published recently.

There are seven different sections to the article. A comprehensive assessment of the relevant literature is given in the second section. The third section lists the research methodology, data-gathering database, tools, and procedures employed in the study. The fourth section explains the bibliometric analysis’s findings. The fifth section presents the article’s conclusion, which includes a discussion of the findings. After elaborating on the potential locations of current research in Section 6, the final section outlined its limits.

## Literature review

### Work-life balance

As stated by Putra (2023), work-life balance, often known as work-life balance, refers to the ability of an individual to manage many demands in their life at the same time in order to meet their obligations to both positions. The common preface is that work-life adjustment arrangements are presented to assist workers in accommodating what they need to do (care) with what they need to do (work). “Work-to-family interference” or “family-to-work interference” are terms used to describe how employees’ employment or workplace conditions can significantly affect their nonwork-related living circumstances and vice versa ( Mache et al., 2023). According to a study on the effects of work-life balance strategies, flexible work schedules may assist women employees regain their equilibrium and relax so they can work freely ( Mohan & Prabha, 2024).

### Police and work-life balance

The term POLICE stands for “Protection Organization for Life and Investment in Civil Establishment”. The word police have been derivative from the Greek word “POLITIA” means the condition of a state or Government. A police job is very hectic in nature and requires physical fitness. The work of the police is to safeguard the public and protect property. The high level of fitness helps the police executives cope with their workload. Police personnel have experienced more conflict between work and personal life due to work pressure, heavy stress, unfixed working hours, etc. Many research studies have found that political pressure, a lack of family time, a negative public image, and low pay are the leading causes of stress among police officers ( SAVITHA, 2023).

### The idea of emotional intelligence

The umbrella term of emotional intelligence (EI), originally initially emerged by Salovey and Mayer (1990), is “the ability to monitor one’s own feelings and emotions, to discriminate among them, and to use this information to guide one’s thinking and action” ( Salovey and Mayer, 1990, p. 189).

### The relationship between work-life balance and emotional intelligence

An unbiased judgment Emotional intelligence (EI) has been separated into four different dimensions by Wong and Law (2002):

1. Emotional expression and evaluation: this dimension characterizes an individual’s understanding of their own emotions and their ability to express themselves honestly.
2. The term “appraisal and recognition of emotions in others” refers to the capacity to see and understand the feelings of people in one’s immediate vicinity.
3. Emotional self-regulation: this pertains to the capacity of individuals to control their emotions, allowing for a quicker recovery from physical discomfort. Using emotions to enhance performance: this refers to the capacity of persons to regulate their feelings.
4. Making use of feelings by channeling them toward productive endeavors and personal achievement. Therefore, emotional intelligence (EI) is a collection of skills that guide and regulate one’s emotions toward their personal as well as professional lives ( Pradhan et al., 2016).

### Assessment of emotional intelligence

Research indicates that EI significantly influences individual performance and work-life balance. For instance, Barsade and Gibson (2007a, b) highlight that affect plays a critical role in organizational dynamics, shaping employee engagement and collaboration. Bell et al. (2012) found that higher EI correlates with reduced job stress and improved employee well-being. The ability to use emotions to solve issues and think more creatively are made possible by emotional intelligence. A critical set of psychological abilities linked to success in general and work-life balance is emotional intelligence, according to Daniel Goleman. Your ability to balance your professional and personal life will depend heavily on your communication, social, and leadership skills ( Ahmad, S.R., Prasad, K.D.V., Bhakuni, S., Hedau, A., Narayan, P.B.S., Parameswari, P., 2023) ( Table 1).

**Table 1.** Definition and views of emotional intelligence.

Variable	Researchers	Publication
Emotional Intelligence	Aaron A. Buchko, 2013 Catherine Prentice, Sergio Dominique Lopes & Xuequn Wang, 2019 Mahima Nandaa, Gurpreet Randhawaa, 2020 Sadaf Naz, Saghir Ahmad, Ayesha Batool, 2021 Azzie T Joyce, Hamrila A. Latif, M Monzer Rahaman, Hridoy Saha, 2021 Rogis Baker, Nur Fatinah Husna Mohamad Puzi, Nur Surayya Mohd Saudi, Haliza Mohd Zahari, Hasimi Sallehudin, Noor Azmi Mohd Zainol, Mohd Nasir Selamat, 2024	Management Research Review Vol. 36 No. 7, 2013 pp. 700-719, DOI: 10.1108/MRR-05-2012-0115 Emotional intelligence or artificial intelligence– an employee perspective, Journal of Hospitality Marketing & Management, DOI: 10.1080/19368623.2019.1647124 Emotional intelligence, work-life balance, and work-related well-being: A proposed mediation model. Colombo Business Journal. 11(2), 1-23. eISSN: 2395-6518, Vol 9, No 2, 2021, pp 141-149 https://doi.org/10.18510/hssr.2021.9214 The Impacts of Emotional Intelligence on Individual Performance and Work-Life Balance: A Conceptual Exploration. International Journal of Academic Research in Accounting Finance and Management Sciences, 11(11), 801–812. Kurdish Studies Jan 2024 Volume: 12, No: 1, pp. 43-57 ISSN: 2051-4883 (Print)|ISSN 2051-4891 (Online)

### The concept of AI

**McCarthy defined AI as “the science and engineering of making intelligent machines”.**

John McCarthy, a computer scientist and emeritus Stanford professor, coined the term “artificial intelligence” (AI) in 1955. AI refers to the simulation of human intelligence by a system or a machine. The aim of artificial intelligence (AI) is to create a machine that can think and behave like a human, including seeing, thinking, learning, planning, predicting, and so forth.

Although artificial intelligence (AI) has been a hot topic for decades, the literature has yet to agree on a definition. The lack of a concept to support empirical research on AI has resulted in a fundamental issue with recognizing AI as a whole. C. Geyer, J.C. Weyerer, and B.W. Wirtz (2019).

“The ability to communicate, acquire knowledge, adapt, and resort to information from experiences, as well as to deal with uncertainty” is how Legg and Hutter (2007) define intelligence in their comprehensive approach.

**The impacts of AI on the equilibrium between work and personal life.**

Task allocation, employee autonomy, and job intensity have all been studied in relation to AI-driven automation by Demerouti et al. (2019) and Bosch et al. (2020). AI’s potential to boost job satisfaction is evident in its ability to reduce mundane work aspects, allowing employees to focus on more meaningful tasks. This shift improves job performance and satisfaction, particularly when employees perceive that AI technologies support their work-life balance ( Kumar, Patel, & Singh, 2024). Furthermore, according to Deloitte, 82% of workers think that using AI technology will enhance their productivity and job satisfaction [2023].

The process of digitalization is socio-technical and involves utilizing digitization potential to accomplish the organization’s strategic and operational goals, which improve WLB ( Strohmeier, 2020). 90% of workers believe or strongly agree that AI has successfully increased organizational equity and, consequently, project performance, according to an IBM research report [ Zhang, H.; Feinzig, S.; Raisbeck, L.; Mccombe 2023].

The possible negative effects on human development are a concern as technology developments, especially in the area of artificial intelligence (AI), change societies [ Y. Qin, Z. Xu, X. Wang, M. Skare, 2023]. Challenges brought about by AI’s impact on employment markets, economic activity, and social structures include biases in AI systems, job displacement, and the creation of a digital divide. Making sure AI development complies with moral principles and enhances human welfare is a challenge for policymakers. With its important role in sustainable economic growth being more widely acknowledged across sectors and attracting the attention of corporations, academia, and government, artificial intelligence (AI) is emerging as a crucial technical instrument for everyday assistance in social and economic activities [ F. Heylighen, 2017]. According to research, artificial intelligence (AI) promotes economic expansion [ P. Aghion, B.F. Jones, C.I. Jones, 2018].

Workflow management and employee productivity have been greatly impacted by artificial intelligence in a variety of businesses ( Vrontis et al., 2022). Qu et al. (2024) claim that such insights and suggestions from AI technologies could enhance an organization’s operations with regard to innovation, decision-making, and enhancing workplace efficiency generally ( Table 2).

**Table 2.** Definition and views of artificial intelligence.

Variable	Researchers	Publication
Artificial Intelligence	Vijay Pereira, 2021 Nishtha Malik and Shalini Nath Tripathi, 2021 Xu, G.; Xue, M.; Zhao, J., 2023 Prof. Smita N. Gambhire1, Dr. Avinash A. Dhavan, Dr. Pritam Kothari, Dr. M.K. Patil, 2023 Patrick Mikalef, Manjul Gupta, 2023 Yasmine Ezzeddine, Petra Saskia Bayerl & Helen Gibson (2023) Tran Minh Tung. (2024)	Human Resource Management Review, https://doi.org/10.1016/j.hrmr.2021.100857 International Journal of Manpower, AI and industry 4.0 led organizations DOI: 10.1108/IJM-03-2021-0173 The Relationship of Artificial Intelligence Opportunity Perception and Employee Workplace Well-Being: A Moderated Mediation Model. Int. J. Environ. Res. Public Health 2023, 20, 1974. https://doi.org/10.3390/ijerph20031974 Artificial intelligence applications – its effects on work-life Balance of employees (a case of precision camshafts limited, solapur) Vol. 72, Issue 01, No. 02, 2023 Artificial intelligence capability: Conceptualization, measurement calibration, and empirical study on its impact on organizational creativity and firm performance https://doi.org/10.1016/j.im.2021.103434 Safety, privacy, or both: evaluating citizens’ perspectives around artificial intelligence use by police forces Kurdish Studies Jan 2024 Volume: 12, No: 1, pp. 43-57 ISSN: 2051-4883 (Print)|ISSN 2051-4891 Opening Up The Workplace: The Way Ai Tools Are Changing Productivity. *Educational Administration: Theory and Practice*, *30*(3), 480–491. https://doi.org/10.53555/kuey.v30i3.1300

### AI in police work

In recent years, artificial intelligence in law enforcement has become an important aspect of police work globally. As AI based police technology becomes increasingly essential to law enforcement, areas like crime prevention and prediction are going through major changes. Several policing techniques have undergone major changes in the name of public safety, with predictive policing being just one outcome of this shift.

### AI in several important ways

**Facial Recognition:** According to reports, AI in law enforcement can identify faces more precisely than humans and save officers time. Machines are able to distinguish faces based on parameters that go beyond what humans can often notice. Facial recognition technology is crucial for police forces. Police personnel use facial recognition and picture data to find missing persons and fugitives.

**Cameras:** In addition to using facial recognition on these photos, AI is also able to recognize objects and intricate events like auto accidents. For law enforcement officials attempting to keep an eye on major events, like music festivals or marathons, object identification is particularly crucial. Drone cameras are also being used by law enforcement organizations, enabling them to conduct faster search and rescue operations and cover more ground.

**Policing using Prediction:** The ability to anticipate crime scenes, the perpetrators, the types of crimes, and the victims is known as AI predictive policing. Police departments and businesses are only now beginning to experiment with predictive policing technology. Eventually, these systems may offer notable advancements in crime prediction and, preferably, prevention.

**Non-violent Crimes:** Artificial Intelligence (AI) has the potential to detect non-violent crimes such as fraud and money laundering because it is adept at seeing patterns that don’t match. By analyzing photographs and spotting details that the human eye would miss, artificial intelligence (AI) can detect counterfeit goods and currencies with a high degree of accuracy.

**Pre-trial Release & Parole:** During the pre-trial stage of the criminal justice system, AI is utilized to decide an offender’s parole terms. By evaluating intricate data sets, these AI systems determine whether an offender should be released on parole and the likelihood that they will flee. Because AI doesn’t make mistakes like people do, it promises to be more efficient than humans at helping.

Because AI eliminates the need for labor-intensive duties, officers may focus on more difficult jobs. Additionally, AI may solve crimes that would otherwise go unnoticed and apprehend offenders who would otherwise escape punishment.

**Figure 1.**
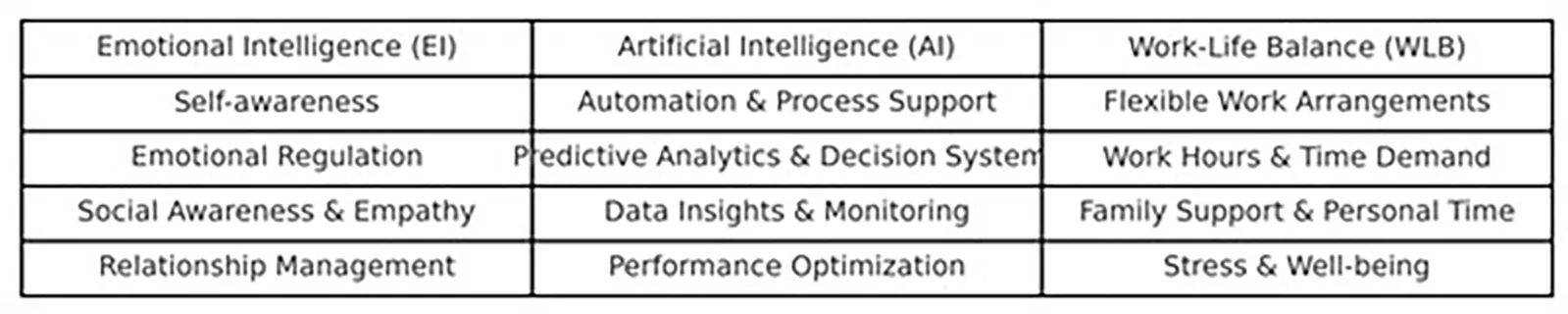
Categorization of EI, AI and WLB factors.

Bibliometric analysis is a research technique that employs quantitative and statistical methods to examine research patterns within a specified period. It has become a prominent method for evaluation, and systematic literature review (SLR) follows a structured, transparent, and replicable process ( Zupic & Cater, 2015). This study utilizes an SLR based on bibliometric analysis to explore the current research landscape on Intelligence and Work-Life Balance, providing a comprehensive overview and identifying future research opportunities.

A systematic search was conducted using academic databases such as Google Scholar and Scopus. The process involved identifying appropriate search keywords, selecting relevant search engines, and extracting studies based on predefined inclusion criteria. Research papers selected for this study were analyzed using bibliometric methods, which have been widely applied in management research ( Kataria et al., 2020). Bibliometric analysis enables researchers to assess intellectual contributions and gain a comprehensive understanding of a research domain. Bibliographic mapping techniques helped identify key research articles, influential authors, and major thematic areas in this field.

This study specifically examines Intelligence and Work-Life Balance, with a focus on police personnel, using VOSviewer software to construct and visualize bibliometric networks. These networks, built through citation analysis, bibliographic coupling, and co-citation techniques, help investigate publication patterns, the knowledge base, and the impact of research articles.

To refine the scope, previous research papers were examined to identify relevant keywords. The selected search terms included “Intelligence,” “Intelligence AND Work-Life Balance,” “Work-Life Balance,” and “Work-Family Balance.” Work-Life Balance (WLB) is an evolving concept that broadens discussions beyond gendered perspectives, promoting a more inclusive approach ( Akanji et al., 2020).

**Figure 2.**
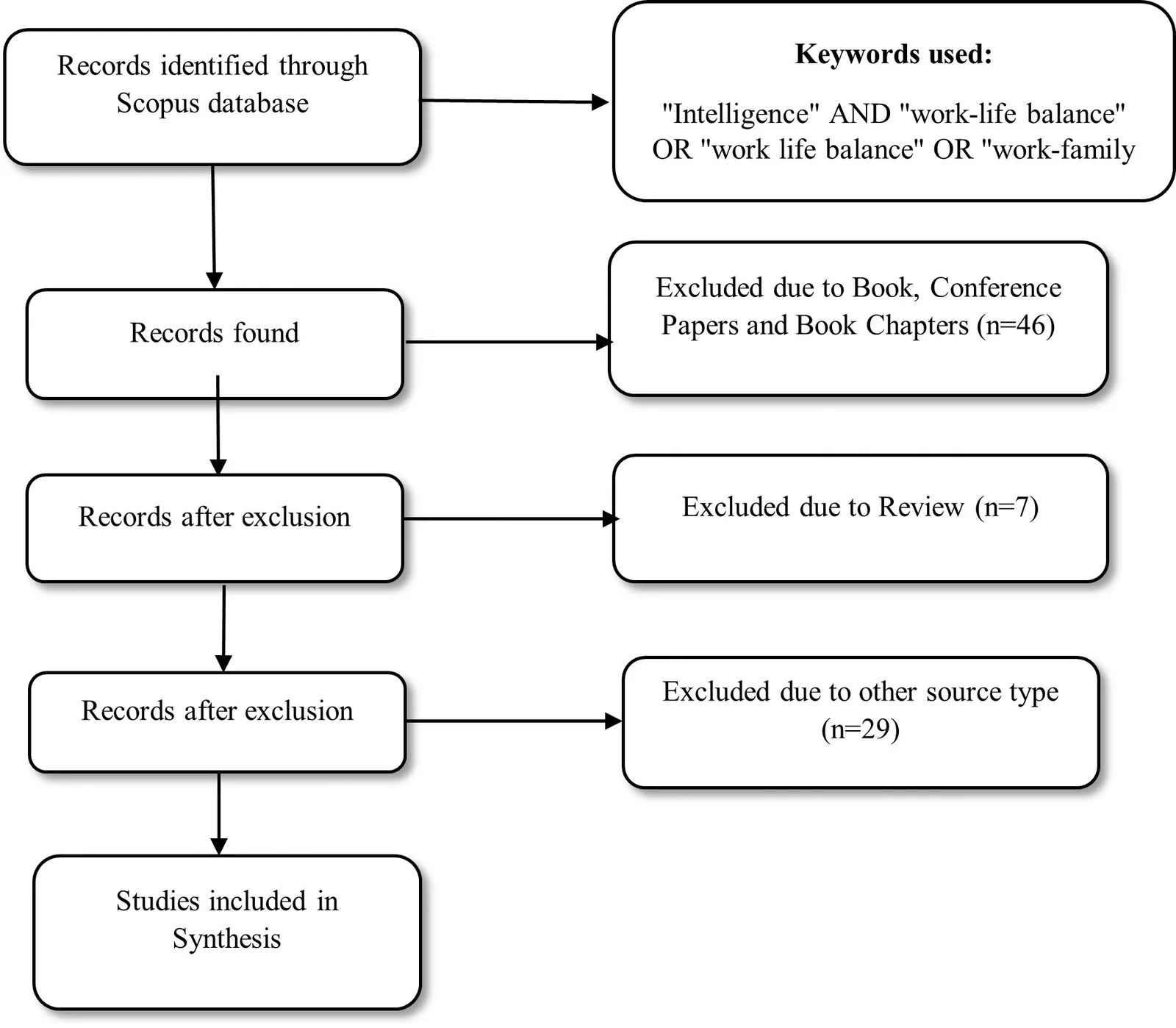
PRISMA flowchart showing screening and exclusion process.

The research utilized bibliometric techniques, employing VOSviewer software alongside Scopus’s analytical functions to analyze search results (Purnomo, Septianto, et al.; Purnomo, Rosyidah, et al.). Bibliometric reviews are a specialized form of systematic reviews that offer a transparent, unbiased, and empirically grounded method for examining knowledge production patterns within specific disciplines (Hallinger & Kovacevic, in press; Zupic & Cater, 2015). This approach is particularly valuable in identifying influential authors and helping scholars recognize existing knowledge gaps ( Aria & Cuccurullo, 2017).

Unlike traditional review methods—such as integrative, scoping, and meta-analysis—that synthesize findings from a limited number of studies, bibliometric reviews analyze bibliographic data from a broader set of research documents ( Zupic & Cater, 2015). Software tools enable this expansive analysis, uncovering overarching trends in knowledge production over time (Hallinger & Kovacevic, in press).

This study developed a comprehensive bibliometric network, visualized using VOSviewer, incorporating elements such as researchers, publication volume, academic affiliations, geographic distribution, research areas, keywords, and author collaborations (Natakusumah). This visualization provides a clearer understanding of the field’s dynamics and key contributors.

## Analysis and results

An overview of the chosen research articles, sample statistics, theoretical underpinnings, keyword analysis, citation, and bibliographic analysis are presented at the start of this section. In order to offer a thorough, critical, and impartial analysis of the cohesive body of evidence, the Intelligence and Work-Life Balance theme research has also been completed.

### Annual trends in publications

**Figure 3.**
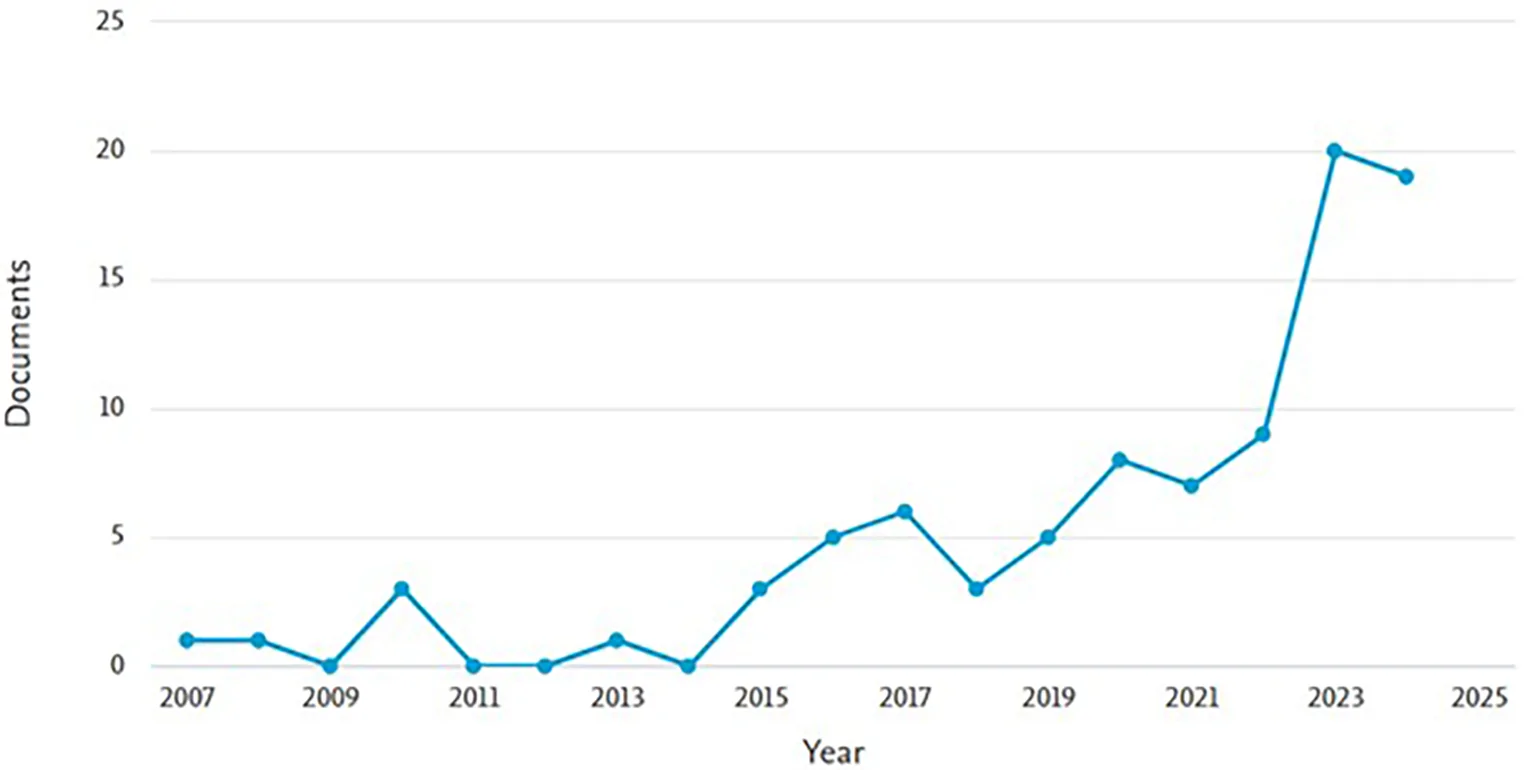
Annual publication trends.

From Figure 3, it can be seen that the increase of international academic document publications in the Intelligence & Work-life balance area has increased every year. The peak of the publication of international academic documents in the field of Work-life balance Innovation was the highest in 2023 with 20 documents. The first paper was published in 2007. Up to the year 2008, paper published are in single digit, but from 2015 it gained momentum and in 2024 the growth almost doubled from 9 publications in 2022 to 19 publications in 2024.

### Document per year by source

**Figure 4.**
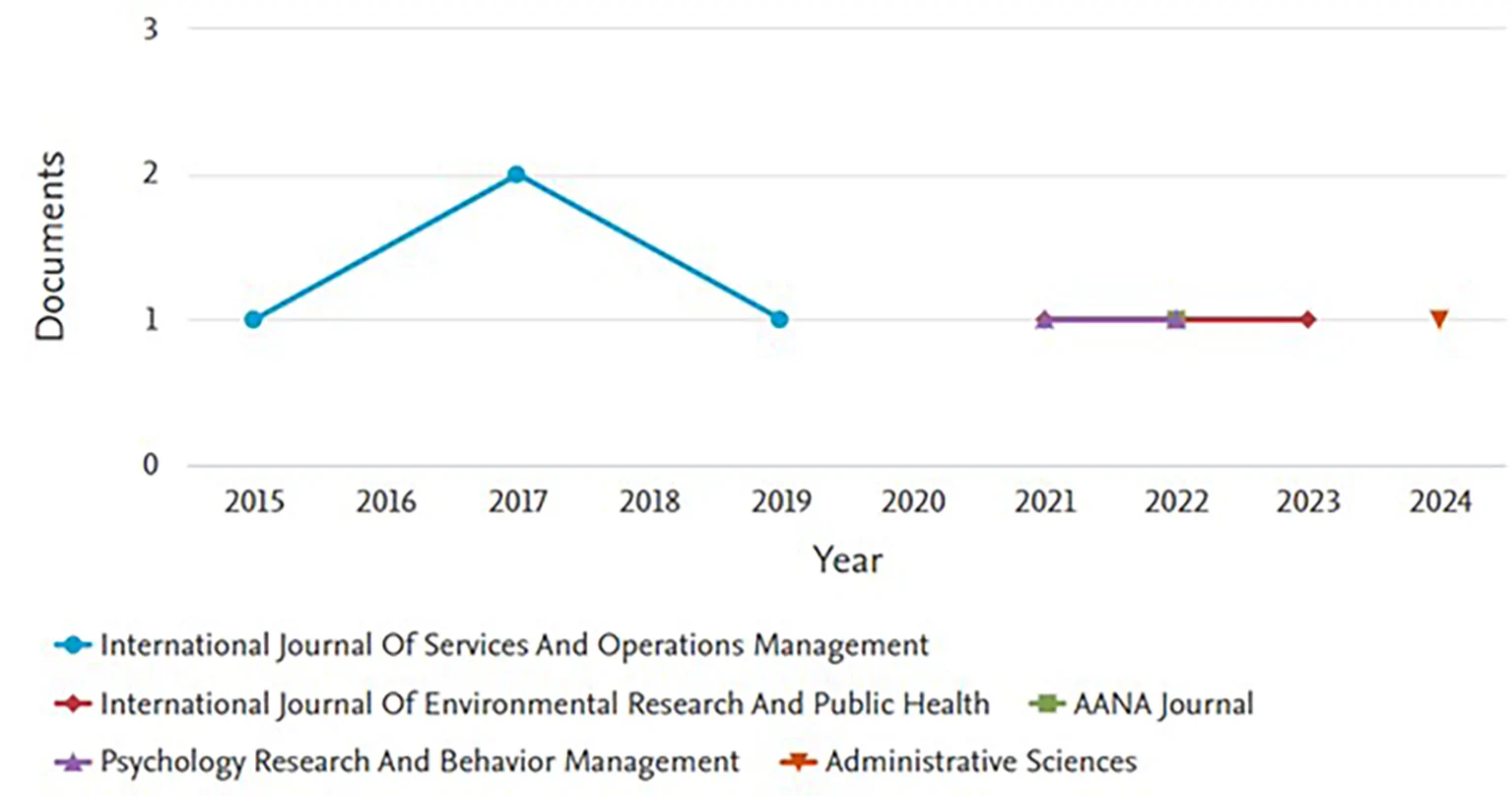
Document distribution by source.

In the annual number of sources of Intelligence & Work-life balance publications is “International Journal Of Services And Operations Management” with 4 documents. Followed by “International Journal Of Environmental Research And Public Health” with 2 documents, “Psychology Research And Behavior Management” with 2 documents, “AANA Journal” and “Administrative Sciences” with 1 document, as shown in Figure.

### Distribution by nation

**Figure 5.**
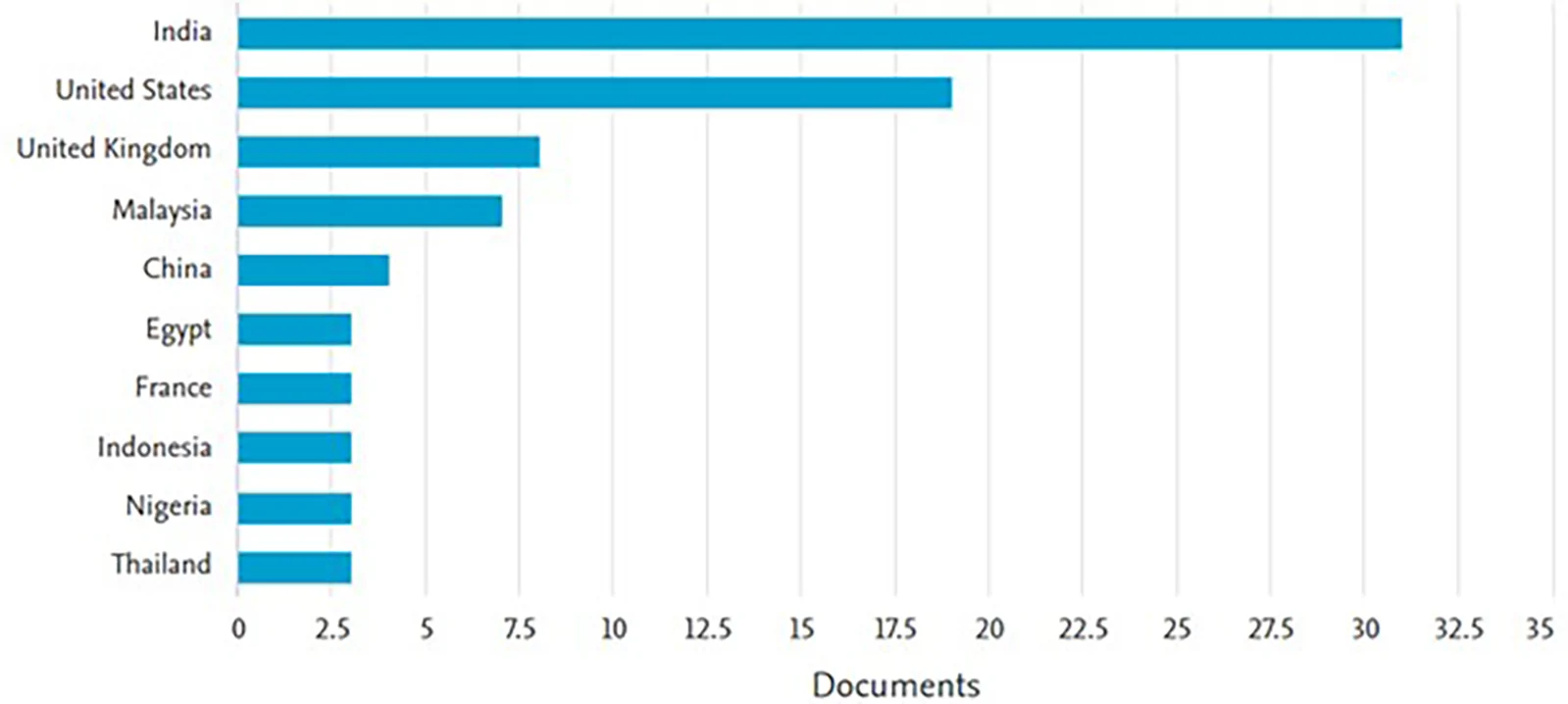
Country-wise publication distribution.

India with 31 academic documents was the leading research nation, Then, with 19 articles, the United States followed, United Kingdom with 8 documents, Malaysia with 7 documents, China with 4 documents, Egypt with 3, France with 3 Nigeria and Thailand with 3 documents.

### Intelligence & Work-life balance publications most individual researcher

**Figure 6.**
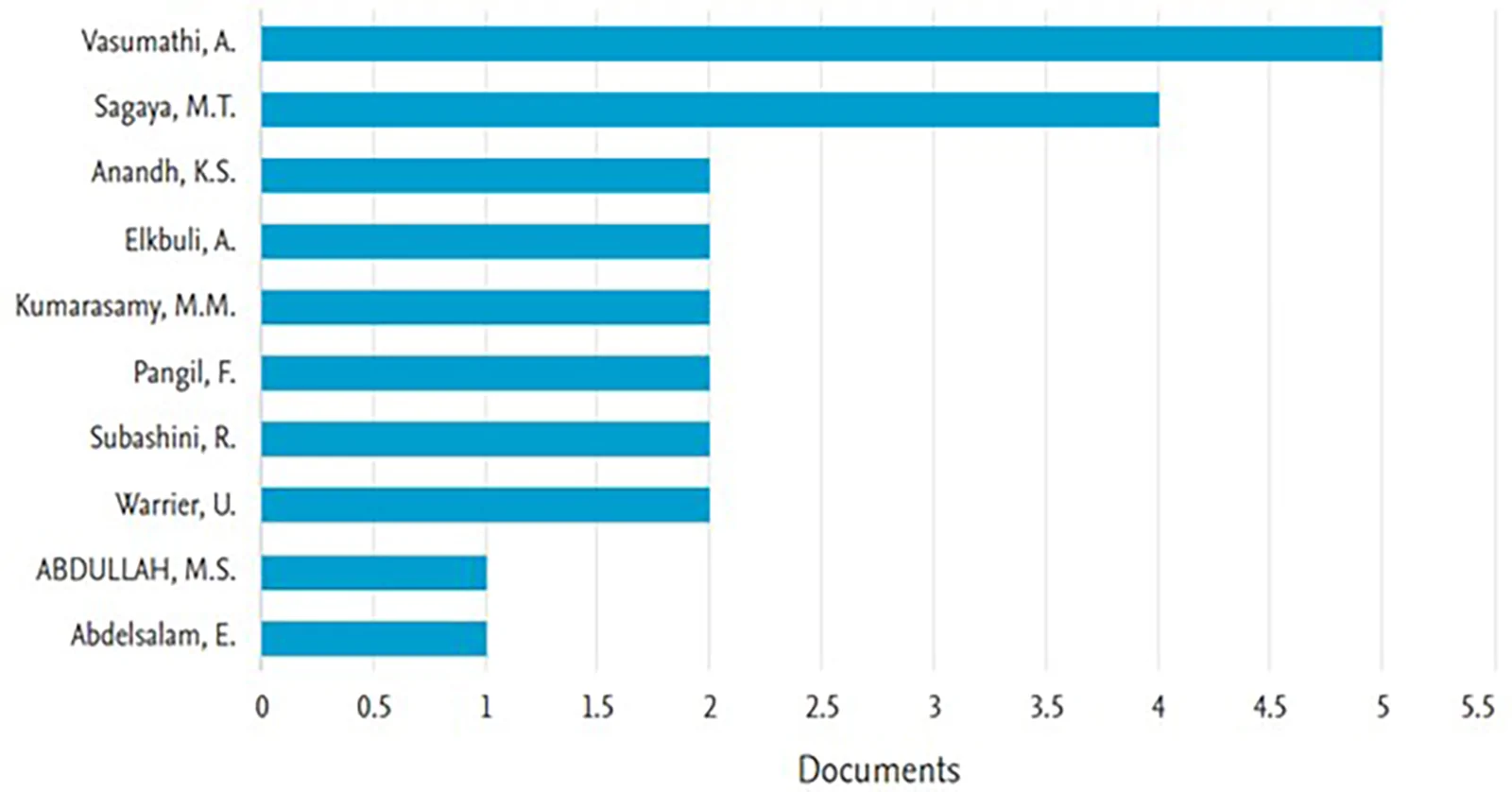
Most productive researchers.

The researcher in the area of Intelligence & Work-life balance to the most writings was Vasumathi, A. with 5 documents. Pursued by Sagaya, M.T. with 4 documents, Anandh, K.S. with 2 documents, Elkbuli, A. with 2 documents, Kumarasamy, M.M., with 2 documents, Pangil, F. with 2 documents and Subashini, R., Warrier, U., with 2 documents, ABDULLAH, M.S., and Abdelsalam, E., with 1 document each.

### The largest frequency of publication of intelligence & work-life balance by subject area

**Figure 7.**
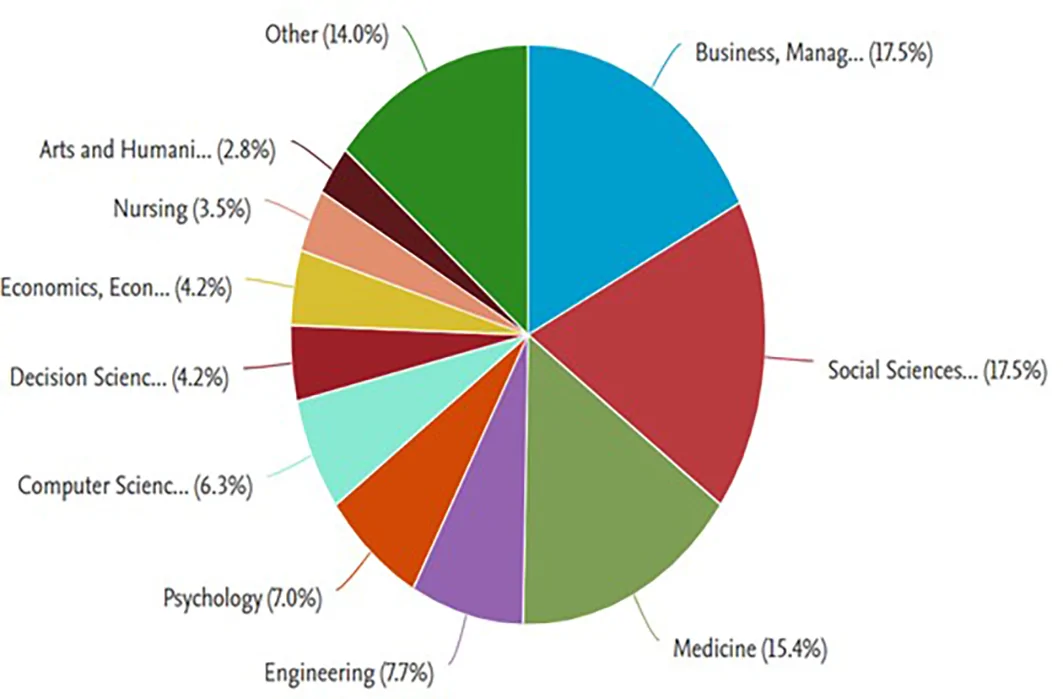
Subject-area distribution.

Among the subjects most frequently seen in international publications on intelligence and work-life balance, business, management, and accounting accounted for 17.5 percent, or 25 documents. The next most common categories are Social Science (17.5%), Medicine (15.4%), Engineering (7.0%), Psychology (7.0%), Computer Science (6.3%), Decision Sciences and Economics, Econometrics, and Finance (4.2%), Nursing (3.5%), Arts and Humanities (2.8%), and Others (14.0%).

### Most common research on sustainable leadership by type of document

**Figure 8.**
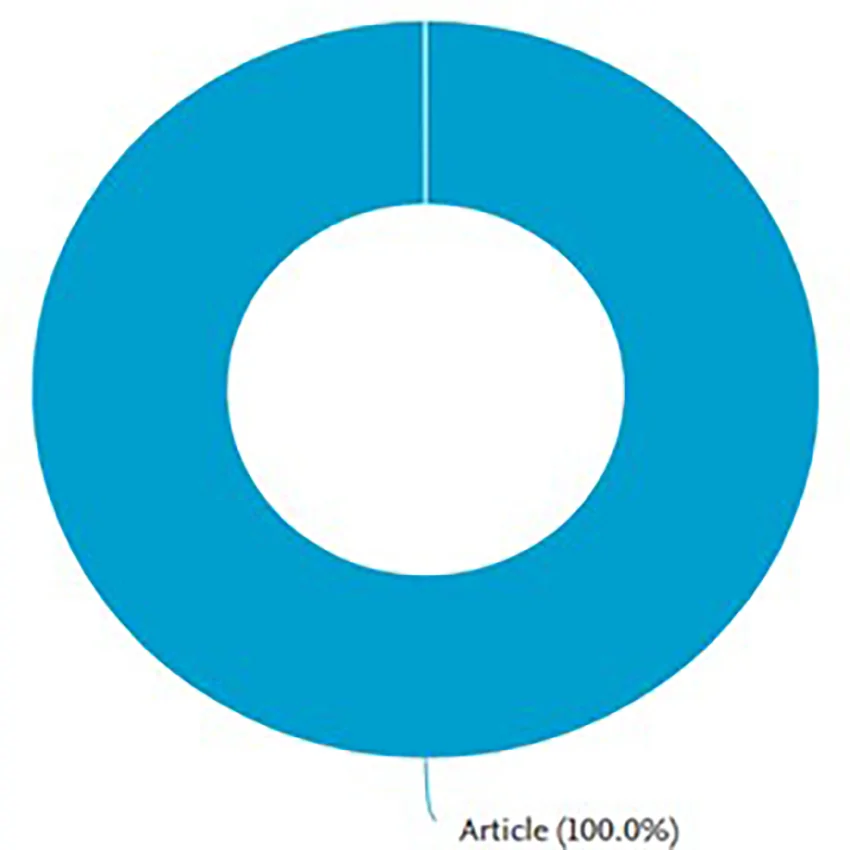
Document type distribution.

The Largest Frequent Document Scientific Type in Intelligence and work-life Balance Publication is Article (100%) with 91 documents ( Table 3).

**Table 3.** Keyword occurrence and link strength.

Sr. No	Keyword	Occurrences	Total link strength
1	Human	29	149
2	Work-life balance	42	141
3	Article	23	131
4	Humans	23	126
5	Female	17	112
6	Emotional Intelligence	49	106
7	Male	16	106
8	Adult	14	98
9	Psychology	10	63
10	Questionnaire	7	54

As seen in Figure 8, we used the author keywords to do a co-occurrence analysis and found seven major clusters of work-life balance and intelligence (EI & AI) literature. The requirement for the minimal quantity of documents relating to the keyword was five repetitions. Thus, out of 901 keywords, 31 keywords met the requirements.

An examination of research on work-life balance and intelligence (EI & AI) shows that three keywords are prevalent: article, work-life balance, and human, as shown in Figure 8.

Keyword network diagrams assist researchers in identifying barren research areas.

**Figure 9.**
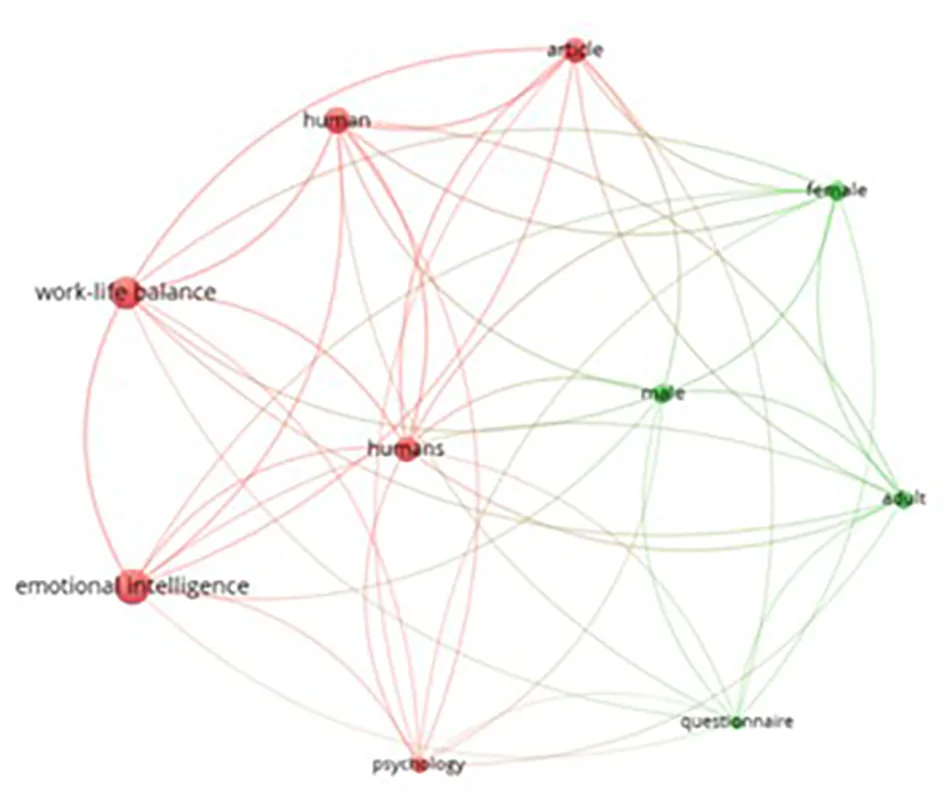
Keyword co-occurrence network.

Density visualization

We conducted the co-occurrence analysis using the author keywords and identified five main density visualizations of Intelligence (EI & AI) and work-life balance literature, as shown in Figure 9. Five repetitions were the criterion for the minimum number of keyword-related documents. Therefore, 5 keywords among 339 reached the thresholds.

Figure 9 illustrates the prevalence of five terms in an examination of research on intelligence (EI & AI) and work-life balance: emotional intelligence, artificial intelligence, work-life balance, and work-life balance.

**Figure 10.**
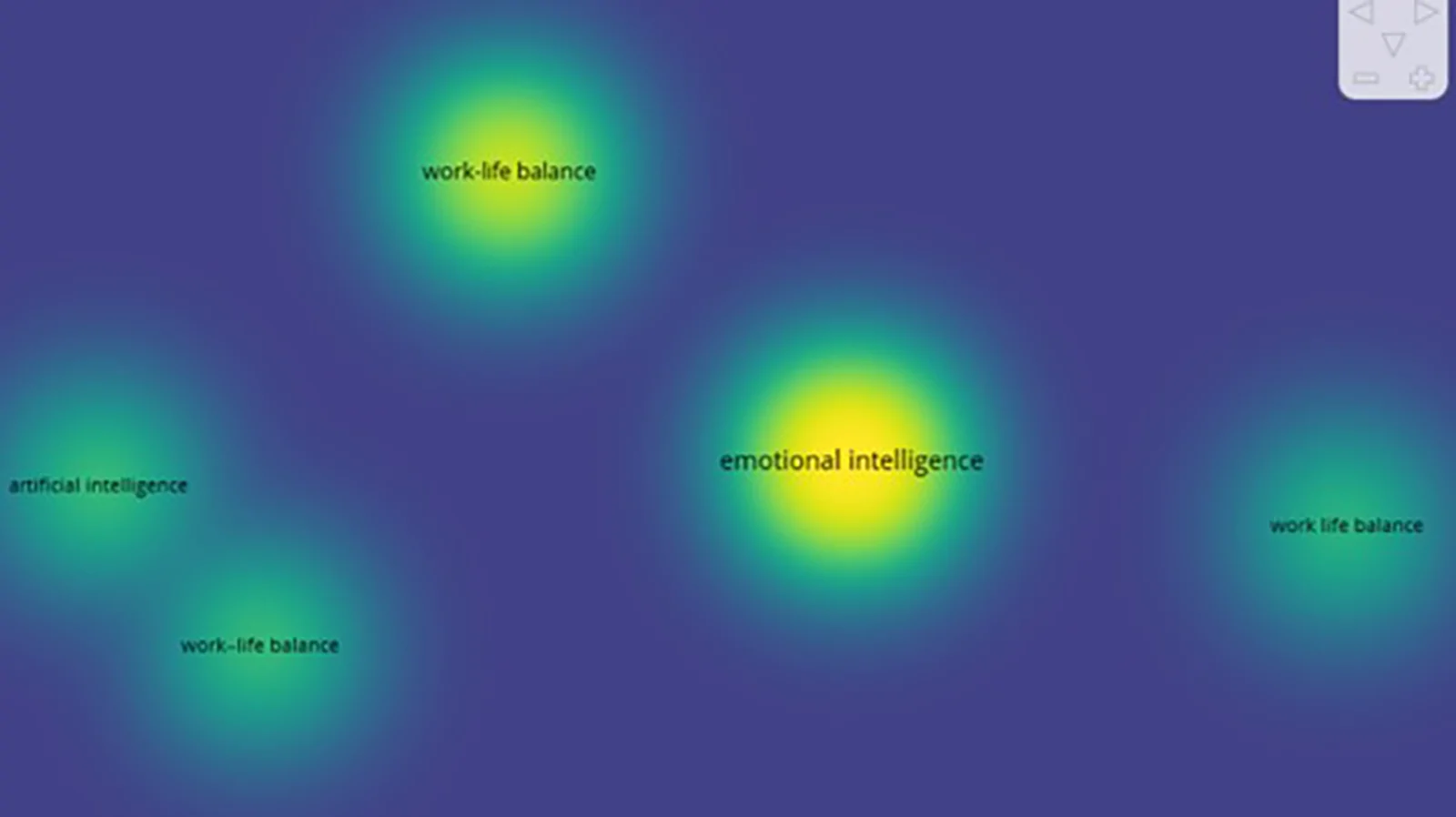
Density visualization.

### Co-authorship analysis

This study has identified the major contributors, academic institutions, and international partners involved in significant collaborative efforts in the field of intelligence (EI & AI) & work-life balance, as represented in network visualizations, by employing co-authorship analysis. This study of co-authorship has targeted the researchers who have the most collaborative engagement in the field of work-life balance and intelligence (EI & AI). Due to this thorough examination, it was determined that 296 authors were covered by this scope. 9 researchers clearly met the predetermined threshold when the condition of at least two publications and citations was used. The network map presented in Figure 9 above shows the names of scholars who engage in the most active collaborations with one another.

With five published publications and twenty-one citations, Vasumathi, A. is without a doubt the most productive researcher in the subject of intelligence (EI & AI) & work-life balance leadership, as shown in Table 2. Sagaya, Mary T., who has received 17 citations and 4 published articles. Later, other well-known scholars in the topic include Subashini, R., Anandh, K.S., Soundarya Priya, M.G., Kumarasamy, and Mokana Muthu, in descending order. They each contributed two published articles and have amassed 11, 3, 3, and 15 citations, respectively ( Table 4).

**Table 4.** Co-authorship metrics.

Sr. No	Author	Documents	Citations	Total link strength
1	Vasumathi, a.	5	21	6
2	Sagaya, mary t.	4	17	5
3	Subashini, r.	2	11	3
4	Anandh, k.s.	2	3	2
5	Soundarya priya, m.g.	2	3	2
6	Kumarasamy, mokana muthu	2	15	1
7	Pangil, faizuniah	2	31	1
8	Elkbuli, adel	2	7	0
9	Warrier, uma	2	20	0

### Co-citation analysis

Co-citation analysis is a novel technique used to understand the cognitive structure of a scientific field. This method of analysis involves recording pairs of source articles that are co-referenced within other source articles. The formation of research clusters occurs when particular pairings of publications are co-referenced by several writers. For this study, co-citation analysis of intelligence research entails the development of network maps that illustrate the connections among cited references, source publications, and authors ( Gerçek & Gerçek, 2022).

The network map of co-cited references on intelligence (AI & EI) is determined by applying a minimum citation count threshold of 20.

## Conclusion

By increasing accuracy, productivity, and human abilities, artificial intelligence (AI) has the potential to fundamentally alter the workplace, the research concludes. While acknowledging the need for careful consideration of potential ethical and societal consequences, such as job displacement and the requirement for worker upskilling, it does so in order to encourage the responsible and inclusive use of technology. The integration of Emotional Intelligence and Artificial Intelligence offers organizations a unique opportunity to enhance employee performance and well-being. By leveraging AI tools alongside EI training, organizations can cultivate a more emotionally aware and productive workforce. Future research should focus on exploring the long-term effects of this integration on organizational culture and employee satisfaction.

## Restrictions and prospective research paths

This bibliometric analysis offers a comprehensive grasp of the development of WLB concepts and applications over a two-decade period. However, future researchers may be able to avoid the study’s various shortcomings. Although our study is the most thorough bibliometric analysis for the first ten years of the new century, its scope is restricted to the three research objectives listed in the introduction, a total of 91 journal papers published and included in the Scopus database are the only ones included in the analysis. Additional relevant materials, such as books and book chapters, conference proceedings, and unreviewed articles not included in Scopus, could offer a fresh perspective on the fields of intelligence and work-life balance.

According to the data, the majority of study articles were written by writers who mostly lived in the USA, UK, and Australia, however, a small number of studies revealed authors from many countries working together Research on the causes of this isolation in research productivity is lacking, but new studies can be started to learn more about the motivations behind cooperation and single-country emphasis. Furthermore, our research shows that WLB research is growing slowly in many other countries. Therefore, a global grasp of the effects and variations of WLB practices and their ramifications is either non-existent or discontinuous. Additionally, the results show a substantial disparity in cooperative research between developing and less developed nations, which will help future researchers who start new studies to close the gaps. To further understand the idiosyncrasies of WLB, future study should concentrate on areas that share similar sociocultural characteristics.

First, subjective findings that could have been bolstered by more factual data were generated by the study’s qualitative methodology. Second, it eliminates SCI journals and only two approaches were used: a bibliographic analysis and a systematic evaluation of the literature. Future studies could employ different methodologies. Additionally, the authors argued that consistent conceptualization and operationalization of WLB research depend on adequately executed field experiments to establish causal links between selected variables.

Last but not least, this systematic review has demonstrated the potential of the research on intelligence and work-life balance. Further findings from our evaluation imply that the future research agenda might focus on filling important research gaps.

## Data Availability

All supporting data for this study, including the PRISMA 2020 Checklist and Flowchart, are openly available on Zenodo under a CC0 1.0 Universal (Public Domain Dedication) license. Repository: Zenodo Dataset Title: PRISMA 2020 Checklist and Flowchart for “AI & EI in Policing: A Bibliometric Review of Their Impact on Work-Life Balance” DOI: https://doi.org/10.5281/zenodo.16743068 License: CC0
