# Supplementary material for: Intelligence Beyond Crime: AI, EI, and the Quest for Work-Life Balance in Policing
Source: F1000Res. 2026 Sep 11;15:645. Originally published 2026 Apr 30. [Version 2] doi: 10.12688/f1000research.170760.2 (PMC13662383; doi:10.12688/f1000research.170760.2)
Supplement: Peer review history

# Reviewer report: Reviewer response for version 2

**DOI:** 10.5256/f1000research.209885.r520280

**Published:** 2026-10-15

**Authors:** Magdalena Parcheva

**Affiliations:** 1 Technical University of Varna, Varna, Bulgaria

The article addresses the topic of the work-life balance in a high-risk professional field - the police. Specifically, the aspects concerning the influence of emotional intelligence and artificial intelligence on the work-life balance are examined. The topic of the article is relevant and significant given the high-risk profession of the police officer, posing as a pressing research problem the study of psychosocial risk factors for the police officer profession and the possibilities for their mitigation through adequate managerial and social approaches and measures, as well as through adequate public policies. There is not much research, especially for high-risk professions such as the police profession.

 The authors have taken my initial comments into account. The justification for the research gap addressed by this study has been improved. The research questions are relatively well posed and set a clear scope of the study. The article presents sufficient information about the research methods. Appropriate research methods have been selected according to the research problem. In accordance with the recommendations, a "methodology" section has been included. Methodological and technical comments have been addressed. The analytical section—specifically the analysis of publication citations—has been expanded and improved.

 Conclusion

 The presented article addresses a current and significant theoretical and practically applied problem that is insufficiently researched and for which there is a need for action, both at the level of managerial and social approaches and measures, and at the level of public policies. The research methodology is appropriate. I am of the opinion that the article can be indexed.

Are the rationale for, and objectives of, the Systematic Review clearly stated?

Partly

Is the statistical analysis and its interpretation appropriate?

Partly

If this is a Living Systematic Review, is the ‘living’ method appropriate and is the search schedule clearly defined and justified? (‘Living Systematic Review’ or a variation of this term should be included in the title.)

Not applicable

Are sufficient details of the methods and analysis provided to allow replication by others?

Yes

Are the conclusions drawn adequately supported by the results presented in the review?

Partly

Reviewer Expertise:

Human Resource Management, Workplace Innovations

I confirm that I have read this submission and believe that I have an appropriate level of expertise to confirm that it is of an acceptable scientific standard.

# Reviewer report: Reviewer response for version 2

**DOI:** 10.5256/f1000research.209885.r520279

**Published:** 2026-10-13

**Authors:** Sandhya DS

**Affiliations:** 1 Christ University, Bengaluru, Karnataka, India

Manuscript is approved with minor changes.

 In literature review - the relationship between work-life and emotional intelligence: this concept is not correct. It is the concept of emotional intelligence given by Wong and Law not the relationship b/w WLB and EI. Correct this one. 

 AI in police work  for this literature review Please add citation and reference

 Literature review is well written. 

 Research methodology is also written well. 

 Discussion is also well written, just remove the heading in the discussion of RQ 1 , 2, and 3. 

 Implications has been written according to the mentioned format. 

 Limitations also written well.

Are the rationale for, and objectives of, the Systematic Review clearly stated?

Yes

Is the statistical analysis and its interpretation appropriate?

Yes

If this is a Living Systematic Review, is the ‘living’ method appropriate and is the search schedule clearly defined and justified? (‘Living Systematic Review’ or a variation of this term should be included in the title.)

Yes

Are sufficient details of the methods and analysis provided to allow replication by others?

Yes

Are the conclusions drawn adequately supported by the results presented in the review?

Yes

Reviewer Expertise:

Research  in Work/life  balance, emotional intelligence, systematic literature review.

I confirm that I have read this submission and believe that I have an appropriate level of expertise to confirm that it is of an acceptable scientific standard.

# Reviewer report: Reviewer response for version 2

**DOI:** 10.5256/f1000research.209885.r521517

**Published:** 2026-09-22

**Authors:** Udik Jatmiko

**Affiliations:** 1 Universitas Tulungagung, East Java, East Java, Indonesia

This manuscript presents a systematic literature review and bibliometric analysis of research concerning artificial intelligence (AI), emotional intelligence (EI), and work-life balance (WLB), particularly in the context of policing. The study analyzes 91 publications and uses bibliometric techniques, including VOSviewer, to examine publication trends, subject areas, geographical distribution, authorship, keyword co-occurrence, and research themes. The topic is relevant and potentially valuable for understanding the emerging intersection between technological and human capabilities in the workplace.

 Overall, the manuscript has a clear research focus, but several methodological details require improvement to ensure transparency and reproducibility.

**1. Rationale and objectives – Partly**

 The rationale and research questions are generally stated clearly. However, the manuscript sometimes uses language suggesting that the study examines the *impact* of AI and EI on WLB, whereas the actual analysis is bibliometric and does not establish causal effects.

 Recommendation: Revise the objectives and abstract to clearly state that the study maps and synthesizes the existing literature rather than evaluating causal effects.

**2. Methods and analysis – No**

 The methodology provides a general description of the search and bibliometric procedures, but insufficient information is provided for exact replication. The manuscript should clearly report the exact search string, search date, database used for the final dataset, inclusion/exclusion criteria, screening procedure, duplicate removal, and VOSviewer settings and thresholds.

 There is also some inconsistency regarding the use of Google Scholar and Scopus, which should be clarified. The PRISMA flowchart should provide the number of records at each screening stage more explicitly.

 This is a major issue that must be addressed to make the review scientifically sound.

**3. Statistical analysis and interpretation – Partly**

 The bibliometric methods and use of VOSviewer are generally appropriate. However, bibliometric associations should not be interpreted as evidence of causal relationships. The authors should distinguish between bibliometric findings and conclusions concerning the actual effects of AI or EI on WLB.

 Recommendation: Clarify the analytical procedures and avoid causal interpretations that are not directly supported by the bibliometric analysis.

**4. Conclusions – Partly**

 The main bibliometric findings are generally reflected in the discussion. However, some conclusions and practical implications imply that AI and EI improve productivity, well-being, or work-life balance, although the present study does not empirically test these relationships.

 Recommendation: Revise the conclusion so that it is directly linked to the bibliometric findings and use cautious language when discussing potential effects.

**Overall recommendation**

 The manuscript addresses an important and emerging topic and provides useful bibliometric evidence. However, the search and screening methodology needs to be reported in substantially greater detail, and the interpretation of the findings should be better aligned with the bibliometric design. In particular, clarification of the search strategy, eligibility criteria, screening process, database selection, and analytical settings must be addressed to ensure scientific soundness and reproducibility.

Are the rationale for, and objectives of, the Systematic Review clearly stated?

Partly

Is the statistical analysis and its interpretation appropriate?

Partly

If this is a Living Systematic Review, is the ‘living’ method appropriate and is the search schedule clearly defined and justified? (‘Living Systematic Review’ or a variation of this term should be included in the title.)

Not applicable

Are sufficient details of the methods and analysis provided to allow replication by others?

No

Are the conclusions drawn adequately supported by the results presented in the review?

Partly

Reviewer Expertise:

Management, Human Resource Management, Organizational Behavior, Psychological behaviour, Work-Life Balance, Emotional Intelligence, Artificial Intelligence, and Bibliometric Analysis.

I confirm that I have read this submission and believe that I have an appropriate level of expertise to confirm that it is of an acceptable scientific standard, however I have significant reservations, as outlined above.

## References

[1] : Determinants of Individual Performance of Professional Village Facilitators: The Mediation of Innovative Work Behavior and Moderation of Work-Life Balance in Kediri Regency, Indonesia. *ECONOMICS* .2025;13(2) : 10.2478/eoik-2025-0036179-201 10.2478/eoik-2025-0036

# Response

**Published:** 2026-10-07

**Authors:** Mohd Asif Shah

**Affiliations:** Economics, Kardan University, Kabul, Kabul, Afghanistan

Thank you for the constructive and valuable comments. We have carefully considered the concerns raised and will revise the manuscript to improve methodological transparency and reproducibility. We will also clarify the study objectives, database and search strategy, screening process, bibliometric procedures, and ensure that the discussion and conclusions do not imply causal relationships beyond the scope of the bibliometric analysis. We appreciate the reviewer’s valuable suggestions, which will help strengthen the manuscript.

# Reviewer report: Reviewer response for version 1

**DOI:** 10.5256/f1000research.188257.r495227

**Published:** 2026-08-03

**Authors:** Sandhya DS, Chandrika Reddy P

**Affiliations:** 1 Christ University, Bengaluru, Karnataka, India; 2 Alliance School of Business, Alliance University, Anekal, Bengaluru, Karnataka, India

**Description of the Manuscript:**

 Overall, the manuscript addresses a relevant and timely topic, and the methodology is well written with appropriate justification. The bibliometric review using VOSviewer has also been presented effectively. However, several revisions are recommended to strengthen the manuscript. The introduction requires better coherence, as the connection between paragraphs is not smooth. In particular, the second paragraph begins abruptly with Emotional Intelligence (EI), and the opening sentence should be revised to provide a clearer transition from the discussion on work–life balance. This coherence must be checked throughout the manuscript. Furthermore, the introduction should explicitly establish the relationships between AI and WLB, EI and WLB, and explain these concepts specifically in the context of police officers. The rationale for conducting this study should also be articulated more clearly so that readers understand the research gap and the significance of the study in the introduction itself. Although the methodology is adequately described, the manuscript does not include a distinct Methodology section heading, which should be added to improve its structure. Theoretical background has to be written and the research gap must be mentioned after the literature review. The discussion section has to be written for each objective. Practical and theoretical implications have to be written. Finally, it is recommended that the Limitations and Future Research Directions sections be presented before the Conclusion, as this would improve the logical flow of the manuscript and better highlight the study's contributions and avenues for future research.

**Strengths**

 The article is highly unique as it presents a Systematic Literature Review (SLR) focusing on the role of Artificial Intelligence (AI) in enhancing Work–Life Balance (WLB).

 The study addresses a novel research problem and provides a unique perspective on the relationship between AI and work–life balance.

 The research objectives are well defined and clearly stated.

 The manuscript is logically organized, enabling a smooth flow of ideas throughout the study.

 The manuscript is well presented in terms of English language, tables, figures, and the overall presentation of data.

 The findings have practical applicability and provide value for researchers and practitioners.

**Weaknesses**

 The introduction lacks sufficient literature to adequately establish the background connecting all the variables, problems and research gaps in the contex of police officers.

 The Conclusion should be placed at the end of the manuscript, following the Limitations and Future Research Directions sections.

 The research design should be described more clearly and presented appropriately in the manuscript.

 The discussion section has not been written and requires a more comprehensive comparison of the findings with existing literature for each research objective or hypothesis. This is a major concern that needs to be addressed.

 The manuscript does not adequately discuss the theoretical and practical implications of the study.

 The theoretical background of the study variables has not been sufficiently explained. In particular, the manuscript should clearly discuss the theoretical relationships among the variables and explain how one variable influences another.

Are the rationale for, and objectives of, the Systematic Review clearly stated?

Yes

Is the statistical analysis and its interpretation appropriate?

Yes

If this is a Living Systematic Review, is the ‘living’ method appropriate and is the search schedule clearly defined and justified? (‘Living Systematic Review’ or a variation of this term should be included in the title.)

Yes

Are sufficient details of the methods and analysis provided to allow replication by others?

Yes

Are the conclusions drawn adequately supported by the results presented in the review?

Yes

Reviewer Expertise:

Research  in Work/life  balance, emotional intelligence, systematic literature review.

We confirm that we have read this submission and believe that we have an appropriate level of expertise to confirm that it is of an acceptable scientific standard, however we have significant reservations, as outlined above.

# Response

**Published:** 2026-08-27

**Authors:** Mohd Asif Shah

**Affiliations:** Economics, Kardan University, Kabul, Kabul, Afghanistan

Thank you for this valuable observation. We agree that the transition between the discussion of work–life balance and emotional intelligence was insufficiently developed. We have therefore substantially revised the Introduction to establish a clearer conceptual progression from AI and work–life balance to emotional intelligence and subsequently to the specific context of police officers. We have also strengthened the discussion of the relationships among AI, EI and WLB and clarified the rationale and research gap.

# Reviewer report: Reviewer response for version 1

**DOI:** 10.5256/f1000research.188257.r487143

**Published:** 2026-06-17

**Authors:** Magdalena Parcheva

**Affiliations:** 1 Technical University of Varna, Varna, Bulgaria

The article addresses the topic of the work-life balance in a high-risk professional field - the police. Specifically, the aspects concerning the influence of emotional intelligence and artificial intelligence on the work-life balance are examined. The topic of the article is relevant and significant given the high-risk profession of the police officer, posing as a pressing research problem the study of psychosocial risk factors for the police officer profession and the possibilities for their mitigation through adequate managerial and social approaches and measures, as well as through adequate public policies. The main *research objective* is to map the evolution of the existing research patterns, identify the current research dynamics, and assess the emerging research paradigms in the field.  The *scope of research* is set by the following criteria: topic of the articles, frequency of publication by subject area, and the most popular subjects considered in the context of the relationship under study. In practice, the article examines the influence between three categories: artificial intelligence (AI), emotional intelligence (EI) and work-life balance in policing, with AI and EI being referred to as "intelligence" in the introductory part of the paper.

***Research methods***

 The study is a systematic literature review, which relies on the methods of bibliometric analysis and content analysis of research papers from the Scopus database. За Processing of the results is used VOS viewer software и Scopus’s analytical functions. Based on the keywords: “Intelligence,” “Intelligence AND Work-Life Balance,” “Work-Life Balance,” and “Work-Family Balance, Work-Life Balance,” 91 publications were identified, analyzed and discussed in the study. The PRISMA method for the systematic literature review was applied.

 A brief literature review is conducted according to the concepts used and the research areas, followed by a presentation of the research methodology, the results of the study, limitations of the study, discussion, outlining research gaps and directions for future research. The literature review includes the following aspects: work-life balance, police and work-life balance, emotional intelligence, relationship between work-life balance and emotional intelligence, assessment of emotional intelligence, the concept of AI, impacts of AI on the equilibrium between work and personal life, AI in police work, and the author's categorization of EI, AI and work-life balance factors is made. In conclusion, the article draws conclusions and outlines directions for future research, mainly focused on the parameters: geographical dimensions in the study of the issue, work-life balance practices and the need to address the highlighted disparity in cooperative research between developing and less developed nations, taking into account sociocultural characteristics in future research on the relationship between AI, EI, and Work-Life Balance and its specific manifestations in Policing.

**Evaluation and recommendations**

 The article addresses a current theoretical and practical problem on which there is not much research, especially for high-risk professions such as the police profession. The work life balance is a factor closely related to the quality of working life and in this sense, valuable knowledge about achieving work life balance is provided by the following studies: Stefana, Marciano, Rossi, et.al (2021); Pot, Konigveld, (2009); Fagnani, Gazier (2026); Eurofound (2026). The relevance of the researched issue in the context of the innovation potential of AI is evidenced by the following studies: Piasna (2024); Pot (2024).

 The main objective of the article „Intelligence beyond Crime: AI, EI, and the Quest for Work-Life Balance in Policing“is well set and formulated. It is recommended to refine the justification for the research gap addressed by this study. The article presents an extensive literature review with an explanation of the concepts used and the aspects related to the studied relationship. It would be good to revise this literature review by shortening it and presenting it in the form of a justification of the research problem and to clearly define the research gap and on this basis to pose the research questions. In this regard, studies could be investigated that examine the nature of the relationships between the studied categories AI, EI, Work-Life Balance, e.g. dependent factor/independent factor; positive/negative relationship, which factors moderate the relationship, as well as to examine the specific manifestations for the police profession. Based on this analysis, the research gap could be defined.

 The introductory part indirectly introduces the concept of “ *intelligence*”, combining emotional intelligence and artificial intelligence. It is necessary to indicate in the introductory part that this concept is being introduced and to explain what this concept includes according to the authors, which could be presented in the form of a working definition of the study. The research questions are relatively well posed and set a clear scope of the study.

 The article presents sufficient information about the research methods. Appropriate research methods have been selected according to the research problem. It is recommended to include a section “Research Methodology” in the article and present the research methods in this section. The classical methodology of systematic literature review has been followed, which is widely used by researchers in various subject areas.

 In *methodological terms*, an area for improvement is the definition of the keywords used in the Scopus database search. It is recommended that instead of searching with the keyword “Intelligence”, the keywords “AI” and “emotional intelligence /EI/” be used. The term “Intelligence” in this sense is introduced by the authors and is not used by the other authors. Searching separately would yield more accurate results, which could then be analyzed and interpreted. In *technical terms*, an inaccuracy has been made in the Introduction and on p. 14 it is stated that the systematic literature review was carried out within Scopus, and on p. 8, Scopus and Google scholar are mentioned; it should be specified within which database the research was carried out.

 It could be that the limitations of the study are moved before the analysis and discussion of the results.

 The statistical analysis of the results is appropriate, but there are deficiencies in the substantive analysis and interpretation of the results.

 With the presented analysis of the results of the study, the research objective is partially realized. It is recommended that the covered publications be analysed in terms of content and that thematic areas of research be outlined, derived based on the studies examined. These directions could be interpreted and based on this interpretation, the conclusions from the study and directions for future research could be formulated. This should be understood as a complement to the analysis made in the article.

 A strength of the article is the inclusion of an analysis of the citations of the publications. It is recommended that this analysis be expanded and deepened, taking into account that citations are a kind of reflection of the areas of impact of the publications related to the studied relationship. The conclusions of the study should be refined from the standpoint of achieving a closer connection with the results of the study.

**Conclusion**

 The presented article addresses a current and significant theoretical and practically applied problem that is insufficiently researched and for which there is a need for action, both at the level of managerial and social approaches and measures, and at the level of public policies. The research methodology is appropriate. The article can be indexed after correction of the comments made.

**References**

1. Piasna, A. Job Quality and digitalization. ETUI. Working paper 2024, Brussel, [Online]. Available: https://www.etui.org/publications/job-quality-and-digitalisation
2. Pot, F. Artificial Intelligence and Job Quality. [Online]. Available: https://bridges5-0.eu/artificial-intelligence-and-job-quality/
3. Eurofound (2026), European Working Conditions Survey 2024: Overview report, Publications Office of the European Union, Luxembourg. European Working Conditions Survey 2024: Overview report | Eurofound

Are the rationale for, and objectives of, the Systematic Review clearly stated?

Partly

Is the statistical analysis and its interpretation appropriate?

Partly

If this is a Living Systematic Review, is the ‘living’ method appropriate and is the search schedule clearly defined and justified? (‘Living Systematic Review’ or a variation of this term should be included in the title.)

Not applicable

Are sufficient details of the methods and analysis provided to allow replication by others?

Yes

Are the conclusions drawn adequately supported by the results presented in the review?

Partly

Reviewer Expertise:

Human Resource Management, Workplace Innovations

I confirm that I have read this submission and believe that I have an appropriate level of expertise to confirm that it is of an acceptable scientific standard, however I have significant reservations, as outlined above.

## References

[1] : Composite Indicators to Measure Quality of Working Life in Europe: A Systematic Review. *Social Indicators Research* .2021;157(3) : 10.1007/s11205-021-02688-61047-1078 10.1007/s11205-021-02688-6

[2] : Quality of working life and organizational performance – two sides of the same coin?. *Scandinavian Journal of Work, Environment & Health* .2009;35(6) : 10.5271/sjweh.1356421-428 10.5271/sjweh.1356 19806274

[3] : Labour market and work–life balance.2026; 10.4337/9781035341603.00023177-190 10.4337/9781035341603.00023

# Response

**Published:** 2026-06-24

**Authors:** Mohd Asif Shah

**Affiliations:** Economics, Kardan University, Kabul, Kabul, Afghanistan

We sincerely thank the reviewer for the constructive and encouraging comments. We appreciate the recognition of the relevance and significance of our study. We will carefully consider all suggestions and address the points raised in a revised version of the manuscript. Thank you for your valuable feedback.
